# Cu(bta)(1,10-phen)ClO_4_ copper complex modulates the carcinogenicity of carboplatin in somatic cells of *Drosophila melanogaster*

**DOI:** 10.1590/1678-4685-GMB-2023-0366

**Published:** 2024-08-05

**Authors:** Paula Marynella Alves Pereira Lima, Priscila Capelari Orsonlin, Nayane Moreira Machado, Rosiane Gomes Silva Oliveira, Lorena Polloni, Raquel Pereira Cruz, Janaína do Couto Almeida, Robson José de Oliveira, Wendell Guerra, Thaise Gonçalves Araújo

**Affiliations:** 1 Universidade Federal de Uberlândia, Instituto de Biotecnologia, Laboratório de Genética e Biotecnologia, Patos de Minas, MG, Brazil.; 2 Centro Universitário de Patos de Minas, Laboratório de Citogenética e Mutagênese, Patos de Minas, MG, Brazil.; 3 Universidade Federal de Uberlândia, Instituto de Biotecnologia, Laboratório de Nanobiotecnologia, Uberlândia, MG, Brazil.; 4 Universidade Federal de Uberlândia, Instituto de Química, Uberlândia, MG, Brazil.

**Keywords:** Cancer, chemotherapy, copper complexes, Drosophila melanogaster, metal-based drugs

## Abstract

Chemotherapy stands out as the main systemic treatment strategy against cancer and still faces problems related to multidrug resistance and severe side effects. Copper-based drugs have been widely explored in medicinal chemistry, since copper is an essential metal for physiological activities with antineoplastic effects. In this context, the present study aimed to evaluate the recombinogenic/mutagenic and anticarcinogenic potential of the complex CBP-01 - [Cu(bta)(1,10-phen)ClO_4_] (Hbta = 4,4,4-trifluoro-1-phenyl-1,3-butanedione and 1,10-phen =1,10-phenanthroline) - through the Somatic and Recombination test (SMART) and the Epithelial Tumor Test (ETT) in *Drosophila melanogaster*, compared with carboplatin (CARB) and cisplatin (CIS) effects. According to our results, CARB and CIS induced a high frequency of mutant spots, which was not verified at higher concentrations of CBP-01. In addition, CBP-01 exhibited mutagenic/recombinogenic potential only at the lowest concentration and after biometabolization. Subsequently, in the ETT test, CBP-01 did not demonstrate carcinogenic effect. Lastly, epithelial tumors were identified in flies treated with CARB and CIS, which were modulated by the CBP-01 complex. Therefore, CBP-01 modulates genotoxicity of other compounds and is a promising metal-based drug for the development of a new anticancer agent or for optimization of therapeutic regimens.

## Introduction

Cancer is a worldwide health problem (Giménez-Bastida and González-Sarrías, 2023). Although evolution in treatment strategies have made cancer death rates drop in developed countries, metastasis remains as a clinical challenge (Michaeli et al., 2023). Additionally, a global increase in the number of cancer patients is expected, with 30 million new cases predicted until 2040 (WHO, 2023).

Chemotherapy is the most important systemic strategy against tumor circulating cells (Huang et al., 2022). However, over the years, toxicity and multidrug resistance (MDR) have been shown, limiting therapeutic efficacy (Vasan et al., 2019). The platinum-based compound cisplatin (CIS) is one of the most widely used drug in oncology, together with carboplatin (CARB) (Wani et al., 2016; Rottenberg et al., 2021). Platinum-based compounds have a mutagenic effect, which can increase tumor heterogeneity, contribute to resistance to chemotherapy and induce secondary tumors (Szikriszt et al., 2020). In this context, it is mandatory to develop new compounds, exploring different metals in addition to platinum. 

Metal complexes containing essential metals, such as copper, have shown promising results as anti-cancer compounds (Gowda et al., 2014; Rodríguez-Mercado et al., 2017; Zehra et al., 2014). Copper is a redox-active metal that easily switches from the reduced Cu(I) to oxidized Cu(II) state or vice versa both in conventional bench chemical reactions and in physiological conditions (Zehra *et al.*, 2014). This metal is a catalytic cofactor of cytochrome oxidase and superoxide dismutase (Cobine et al., 2021), and is involved in mitochondrial respiration (Ruiz et al., 2021). Copper complexes can also generate reactive oxygen species (ROS) (Mukherjee et al., 2023), intercalate with DNA (Romo et al., 2021) and induce apoptosis (Ji et al., 2023). Moreover, they may be effective against tumors that are resistant to conventional chemotherapy (Li et al., 2023; Qian et al., 2023). Previously, our group synthesized a Cu(II) complex, [Cu(bta)(1,10-phen)ClO4], containing the deprotonated ligand 4,4,4-trifluoro-1-phenyl-1,3-butanedione (bta) and 1,10-phenanthroline (1,10-phen), called CBP-01 (do Couto Almeida et al., 2015). In murine tumor cells, CPB-01 induced ROS production, DNA damage and apoptosis, inhibiting the cell cycle (Polloni et al., 2019). However, the *in vivo* genotoxicity of copper complexes, especially CBP-01, is unknown, and this information is relevant as they are suggested as potential antineoplastic compounds. In addition, efforts have been made to develop *in vivo* tests seeking alternative models to those of mammals (Pitchakarn et al., 2021).

*Drosophila melanogaster* fly is a eukaryotic organism used for decades to monitor genetic damage caused by chemical agents. It can activate enzymatically pro-mutagens and pro-carcinogens *in vivo*, considered as an optimized model for the detection of mutagenic/recombinogenic activity (Graf et al., 1984; Nepomuceno, 2015). According to Adams et al. (2000), genetic and metabolic similarities between flies and humans reinforce the importance of *D. melanogaster* as an experimental platform for the study of human diseases related to replication, repair pathways, translation and drug metabolism.

*D. melanogaster* is the experimental model for the somatic mutation and recombination testing (SMART) and the Epithelial Tumor Test (ETT). SMART is well described and widely used in toxicology for mutagenic and recombinogenic evaluation of different compounds, including antineoplastic drugs (Singer and Graf, 1992; Danesi et al., 2010; Naves et al., 2018). The ETT test, in turn, detects loss of heterozygosity for the tumor suppressor gene *warts* (*wts*) in *D. melanogaster* imaginal disc cells. Loss of function of this gene triggers increased cell proliferation and epithelial cell hypertrophy, leading to abnormal deposition of extracellular matrix during the fly development (Nepomuceno, 2015). Thus, the test allows evaluating the carcinogenic potential of a substance of interest (Vasconcelos et al., 2017).

In this context, the present study aimed to evaluate the mutagenic/recombinogenic and carcinogenic potential of CBP-01 alone or simultaneously administered with CARB, using SMART and ETT tests in *D. melanogaster.* Importantly, the results for CBP-01 were compared with CARB and CIS. We believe that these results can be useful for the development of new therapeutic strategies, paving a way for innovative treatments besides platinum-based compounds.

## Material and Methods

### Chemical agents

CBP-01 or [Cu(bta)(1,10-phen)ClO_4_] (Hbta = 4,4,4-trifluoro-1-phenyl-1,3-butanedione and 1,10-phen =1,10-phenanthroline) was synthesized and characterized according to our previous work (do Couto Almeida et al., 2015). 

Doxorubicin (DOX), Adriblastina^®^, Pfizer, CAS number 25316-40-9, was used as positive control. The concentration was based on previous studies that demonstrated the induction of homologous recombination in *D. melanogaster* when DOX was used at 0.4 mM (Orsolin et al., 2015; Braga et al., 2018; Lima et al., 2018).

Cisplatin (CIS), CAS number 15663-27-1, was purchased from Sigma-Aldrich^®^ and used at 0.025 mM as previously demonstrated (Danesi et al., 2010; de Campos et al., 2017). The concentration of Carboplatin (CARB) or B-Platin^®^ CAS number 41575-94-4, Blau Farmacêutica, was defined according to De Campos *et al.* (2017) at 0.5 mM.

5% ethanol was used as negative control and for the dilution of the compounds. All dilutions were prepared immediately before use.

### Crossings

*SMART*

Three different strains of *D. melanogaster* were used: (ii) females flr-3 (flr^3^/In(3LR)TM3, ri pp sep l(3)89Aabx^34e^ and Bd^s^; (ii) females ORR;flr3 (ORR; flr3/In(3LR)TM3, ri pp sep l(3) 89Aabx^34e^ and Bd^s^; (iii) and males mwh(mwh/mwh). In the SMART assay, two crosses were performed, according to the methodology proposed by Graf and collaborators (Graf et al., 1984; Graf and van Schaik, 1992):

1. Standard (ST) cross: virgin females *flr* ^*3*^ were crossed with males *mwh*. The descendants have basal levels of cytochrome P450 enzymes for the evaluation of mutagenic agents (Graf et al., 1984).

2. High bioactivation (HB) cross: females *ORR* were crossed with males *mwh*. This crossing results in high levels of P450 promoting greater biotransformation (Graf et al., 1989; Graf and van Schaik, 1992).

Both crosses produced two types of progeny, which were analyzed in this study: the marked trans-heterozygous (MH, *mwh*+/+*flr* ^*3*^ ), with smooth wing edge phenotype, and individuals heterozygous for the *TM3* balancer (BH, *mwh*+/+*TM3*) with the wing having a serrated appearance (Guzmán-Rincón and Graf, 1995). 

Over treatment, substances that damage the fly DNA lead to loss of heterozygosity and expression of recessive genes, giving rise to a clone of mutant cells that can be detected by means of mutant trichomes on the wing of the adult (Guzmán-Rincón and Graf, 1995; Spanó et al., 2001).

*ETT*

Virgin females *wts/TM3, Sb* ^*1*^ and males *mwh/mwh* were paired to obtain heterozygous *wts +/+ mwh* larvae*.* This test evaluates the *warts* marker encoded by the *wts* gene, the *D. melanogaster* homolog of the mammalian tumor suppressor gene *LATS1* (Siam et al., 2009). Deletion of the *wts* gene in the wild type and the consequent expression of the mutant allele lead to the formation of highly invasive cell clones in the imaginal discs of larvae and the development of epithelial tumors in the body and appendages of adult flies. When homozygous, the mutation is lethal. Therefore, the presence of the balancing chromosome *TM3, Sb* ^*1*^ is necessary in crosses (Sidorov et al., 2001).

### Toxicity test

The toxicity (TX) assay was performed in order to establish the concentration of CBP-01 to be used in the SMART and ETT tests. CBP-01 starting concentrations were based on previous studies conducted with compounds with similar properties, such as Casiopeina II-gly and Casiopeina III-Ea (Jiménez et al., 2016; Vidal et al., 2017). 

For the SMART assay, 100 larvae obtained from ST and 50 from HB crossings were counted and placed in separate tubes containing 1.5 g of culture medium (mashed potatoes) for *D. melanogaster* (Spanó et al., 2001) and 5.0 mL of different concentrations of CBP-01 (0.03 mM, 0.06 mM, 0.12 mM, 0.25 mM, 0.50 mM, 1.00 mM, 2.00 mM and 4.00 mM). For the ETT assay, *wts +/+ mwh* heterozygous larvae obtained from crossing virgin females *wts/TM3, Sb* ^*1*^ with *mwh/mwh* males (Nepomuceno, 2015) were counted and placed in tubes containing 1.5 g of culture medium (mashed potatoes) with CBP-01 at the concentrations mentioned above. Negative control (5% ethanol) and ultrapure water were also included to evaluate the toxicity of the compounds.

In both tests, the toxicity of CARB (0.5 mM) and CIS (0.05 mM and 0.025 mM) was evaluated. Egg laying occurred within a period of 8 h. The larvae, resulting from the eggs hatching, were collected using a fine mesh sieve, washed with reverse osmosis water and finally counted. The number of surviving flies for each treatment indicated the toxicity of the compounds. 

### Somatic mutation and recombination test (SMART) in *D. melanogaster*

The SMART test was performed according to the methodology proposed by Graf et al. (1984) and Graf and van Schaik (1992), with modifications. Briefly, after crossings (section 2.2), flies were transferred to a flask containing the hatching medium, a layer of yeast (*Saccharomyces cerevisiae*) and supplementation with sugar under a solid base of agar (4% w/v). Oviposition occurred over a period of 8 h. After 72 h (± 4 h), the third instar larvae were washed and placed in individual vials containing 1.5 g of mashed potato flakes (HIKARI^®^) as described by our group (Spanó et al., 2001) and subjected to chronic treatment for 48 h, until development of the pupal stage. CBP-01 (0.03 mM, 0.06 mM, 0.12 mM and 0.25 mM) diluted in 5% ethanol, CARB (0.5 mM), CIS (0.025 mM), DOX (positive control, 0.4 mM) and 5% ethanol (negative control) were added and tested in two independent experiments, under optimal laboratory conditions (25 ± 4 °C and 65% RH). 

After metamorphosis, the adult flies were transferred to vessels containing 70% (v/v) ethanol. The wings were removed, with entomological forceps, and mounted on coded slides containing Faure solution (30 g of gum arabic, 50 mL of distilled water, 200 g of chloral hydrate and 16 mL of glycerol). The wings (from both the dorsal and ventral surface) were analyzed under a light microscope, at a magnification of 400x (Graf et al., 1984). Frequency and size of single and twin spots were recorded.

### Epithelial tumor test (ETT) in *D. melanogaster*

Egg laying of the descendants of the cross between virgin females *wts/TM3, Sb* ^*1*^ and males *mwh/mwh* occurred over a period of 8 h. Third stage larvae (72 h ± 4 h) were collected, placed in tubes containing 1.5 g of culture medium (mashed potato) for *D. melanogaster* and treated for 48 h (Nepomuceno, 2015) with CBP-01 (0.03 mM, 0.06 mM, 0.12 mM, 0.25 mM), CARB (0.5 mM) or CIS (0.025 mM). Combined treatments were also performed, in which CBP-01 (0.03 mM, 0.06 mM, 0.12 mM, 0.25 mM) was associated with CARB (0.5 mM). DOX (0.4 mM) was used as a positive control and 5% ethanol as a negative control. Treatments were carried out in quadruplicates.

Following metamorphosis, the adult flies were transferred to recipients containing 70% ethanol. Males and females of the (*wts +/+ mwh*) genotype, which express wild hairs (long and thin), were analyzed for tumor presence. Adult flies with the chromosome balancer (*TM3*,*Sb1*), expressed by truncated bristles, were not included. The flies were observed using a stereoscopic magnifying glass and entomological tweezers. Only tumors that were large enough to be unequivocally classified were recorded (Eeken et al., 2002).

### Statistical analysis

Statistical comparisons of survival rates in TX test were performed with the Chi-squared (X^2^) test for ratios of independent samples, using the program GraphPad Prism 8.0 (GraphPad Software Inc., La Jolla, CA, USA), with significance level of p < 0.05.

For the SMART test, the statistical analysis was carried out in accordance with the multiple decision procedure proposed by Frei and Würgler (1988), at a significance level of 5%, resulting in different diagnoses: positive, weakly positive, negative and inconclusive. The frequency of each type of spot (small or large single spot and twin spot), and the total frequency of spots per fly, for each treatment, were recorded. The comparison was made in pairs (CBP-01 *vs* negative control/ CARB *vs* negative control/ CIS *vs* negative control; DOX *vs* negative control; and CBP-01 + DOX *vs* positive control).

The calculation of recombinogenic activity was based on the frequency of induction of mutant spots per 10^5^ cells/division. Comparisons of induction of mutant spots in descendants MH and BH were performed as follows: (i) Frequency of mutation (FM) = frequency of clones in BH individuals/ frequency of clones in MH individuals/ (ii) Frequency of recombination (FR) = 1 - frequency of mutation (FM) (De Rezende *et al.*, 2011). According to Abraham (1994), the percentage of induction of recombination was calculated using the frequency of clones per 10^5^ cells, normalized by the control, as follows: [(DOX alone - CBP-01 + DOX)/DOX alone × 100].

Finally, for the ETT test, comparisons were determined by the non-parametric Mann, Whitney and Wilcoxon U test, with a significance level a = 0.05, using Prophet 5.0 (Phophet Software) (Nepomuceno, 2015).

## Results

### Mutagenic and recombinogenic effects

**Figure 1 -:**
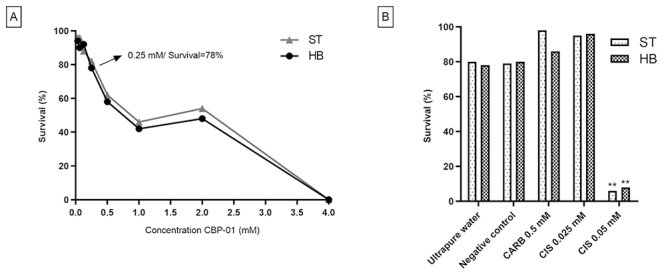
Survival of *D. melanogaster* evaluated after metamorphosis from third-stage larvae. (A) Larvae treated with different of concentrations of CBP-01. (B) Larvae treated with different concentrations of carboplatin (CARB) and cisplatin (CIS). Larvae were obtained from standard (ST) and high bioactivation (HB) crosses in Somatic Mutation and Recombination test (SMART). NC: Ultrapure water. **Statistical difference (p < 0.01) comparing to water control according to the X^2^ test for ratios for independent samples.

At first, the toxicity of CBP-01 was evaluated for the SMART assay. The survival rates are shown in Figure 1, and we observed a dose-dependent response. No statistical difference was found between the negative control (5% ethanol) and ultrapure water. The highest concentration of CBP-01 (4.00 mM) was lethal to all flies, and 0.25 mM of CBP-01 promoted a survival rate over 70% (Figure 1 A ), with no statistical difference when compared to negative control and ultrapure water. In the other concentrations (0.12 mM, 0.06 mM and 0.03 mM), there was a greater survival rate (> 70%), with a significant difference when compared to negative control and ultrapure water. A survival rate within the range of 70% is considered as ideal and non-toxic to *D. melanogaster* (Carmona et al., 2011; Orsolin et al., 2015) and, for this reason, the concentrations 0.03 mM, 0.06 mM, 0.12 mM and 0.25 mM of CBP-01 were chosen for further analyses in SMART. 

Figure 1 B  shows the survival rate of larvae treated with CARB (0.5mM) and CIS (0.025 mM and 0.05 mM). In the treatment with 0.05 mM of CIS, survival was only 6% and 8% in the ST and HB crosses, respectively, being significantly toxic when compared to negative control and ultrapure water. On the other hand, in treatments with 0.5 mM CARB and 0.025 mM CIS, more than 80% of flies emerged in both ST and HB crosses. Therefore, 0.5 mM CARB and 0.025 mM CIS concentrations were used in subsequent SMART assays.

Table 1 shows MH (trans-heterozygous) and BH (balancer heterozygous) descendants of the ST and HB crosses of the SMART test, respectively. Flies were treated only with CBP-01. In ST cross/ MH progenies, CBP-01 did not promote significant difference in the total number of spots when compared to the negative control (p > 0.05). However, in HB/ BH progenies, at the lowest concentration of CBP-01 (0.03 mM), we identified a significant increase in spots when compared to negative control. For this reason, the frequencies of clones observed in the MH and BH descendants treated with 0.03 mM of CBP-01 were compared, in order to check whether the increased spots observed resulted from mutational events or recombinational events. In the MH progeny, mitotic recombination and other mutagenic events may occur. In BH (*mwh/TM3*) descendants, all recombinogenic events are eliminated, since the *TM3* balancer chromosome impedes recombination in these individuals (Spanó et al., 2001). We found that the spots induced by 0.03 mM of CBP-01 in MH progenies were mainly due to recombination (52.15%). 

**Table 1-:** Summary of results obtained in the marked trans-heterozygous descendants (MH) and balancer-heterozygous (BH) of *D. melanogaster* derived from the standard cross (ST) and high bioactivation cross (HB). Flies were treated with different concentrations of CBP-01**.** Doxorubicin (DOX) at 0.4 mM was used as positive control and the diluent (5% ethanol) was used as a negative control.

Treatments		N^º.^ of flies (N)	Spots per fly (n^º.^ of spots) statiscal diagnosis^a^												Spots with mwh clone^c^ (n)	Mean clone size class^c,d^ (î)	Frequency of formation / 10^5^ cells per cells divisiond		Recombination (%)
DOX (mM)	CBP-01 (mM)		Small single (1-2 cels)^b^ m = 2			Large single (>2 cels)^b^ m = 5			Twin m = 5			Total spots m = 2					Observed	Control corrected	
mwh/flr3 (MH)																			
Cross ST																			
0	0	60	0.37	(22)		0.05	(3)		0.00	(0)		0.42	(25)		25	1.40	0.56		
0.4	0	60	0.48	(29)	i	0.92	(55)	+	0.80	(48)	+	2.20	(132)	+	110	3.42	18.06	18.86	
0	0.03	60	0.38	(23)	-	0.03	(2)	i	0.05	(3)	i	0.47	(28)	-	26	1.85	0.80	0.00	
0	0.06	60	0.32	(19)	-	0.07	(4)	i	0.02	(1)	i	0.40	(24)	-	24	2.13	0.89	0.00	
0	0.12	60	0.23	(14)	-	0.07	(4)	i	0.02	(1)	i	0.32	(19)	-	19	1.79	0.56	0.10	
0	0.25	60	0.12	(7)	-	0.07	(4)	i	0.02	(1)	i	0.20	(12)	-	12	2.75	0.69	0.16	
Cross HB																			
0	0	60	0.78	(47)		0.17	(10)		0.00	(0)		0.95	(57)		57	2.00	1.95		
0.4	0	60	1.52	(91)	+	1.98	(119)		0.25	(15)	+	3.75	(225)	+	222	3.25	18.06	18.12	88.98
0	0.03	60	1.35	(81)	+	0.05	(3)	-	0.03	(2)	i	1.43	(86)	+	86	1.51	2.09	0.36	52.15
0	0.06	60	0.83	(50)	-	0.18	(11)	i	0.00	(0)	i	1.02	(61)	-	60	1.77	1.77	0.00	
0	0.12	60	0.82	(49)	-	0.10	(6)	-	0.03	(2)	i	0.95	(57)	-	57	1.70	1.58	1.58	
0	0.25	60	0.60	(36)	-	0.07	(4)	-	0.02	(1)	i	0.68	(41)	-	41	1.85	1.27	0.71	
mwh/TM3 (BH)																			
Cross HB																			
0	0	30	0.33	(10)		0.03	(1)		f			0.37	(11)		11	1.36	0.48		
0.4	0	30	0.93	(28)	+	0.17	(5)	i				1.10	(33)	+	33	1.82	1.99	1.55	
0	0.03	30	0.73	(22)	+	0.07	(2)	i				0.80	(24)	+	24	1.29	1.00	0.52	

Marker-trans-heterozygous flies (mwh/flr3) and balancer-heterozygous flies (mwh/TM3) were evaluated.

^a^ Statistical diagnoses according to Frei and Würgler (1988, 1995): +, positive; -, negative; i, inconclusive. m = multiplication factor for significantly negative results. Level of significance P ≤ 0.05.

^b^ Including rare flr3 single spots.

^c^ Considering mwh clones from mwh single and twin spots.

^d^ Frequency of clone formation: clones/flies/48,800 cells (without size correction) Frei *et al.* (1992).

^f^ Only mwh single spots can be observed in heterozygous individuals mwh/TM3, since the balancer chromosome TM3 does not contain the mutant gene flr3.

DOX was used and positive control and, when compared to the negative control, induced significant frequency of spots, as expected (Table 1). Through the comparison between the clones of MH and BH individuals, DOX mainly induced recombination (88.98%). 

Table 2 summarizes the results for the treatments with CARB (0.5 mM) and CIS (0.025 mM) for ST and HB crosses. MH progeny can also be visualized. When compared to the negative control, both had a high frequency of spots, showing their mutagenic / recombinogenic effects. Moreover, we found that spots induced by CARB and CIS were mainly due to recombination (66.66% and 86.71% in ST cross; 67.16% and 86.98% in HB cross, respectively).

**Table 2 -:** Summary of results obtained in the marked trans-heterozygous descendants (MH) and balancer-heterozygous (BH) of *D. melanogaster* derived from the standard cross (ST) and high bioactivation cross (HB) treated with Carboplatin (CARB) (0.5 mM) and Cisplatin (CIS) (0.025 mM). Diluent (5% ethanol) was used as negative control.

Treatments		N^0^. of flies (N)	Spots per fly (n^0^. of spots) statiscal diagnosis^a^												Spots with mwh clone^c^ (n)	Mean clone size class^c,d^ (î)	Frequency of formation / 10^5^ cells per cells divisiond		Recombination (%)
CARB (mM)	CIS (mM)		Small single (1-2 cels)^b^ m = 2			Large single (>2 cels)^b^ m = 5			Twin m = 5			Total spots m = 2					Observed	Control corrected	
mwh/flr3 (MH)																			
Cross ST																			
0	0	60	0.37	(22)		0.05	(3)		0.00	(0)		0.42	(25)		25	1.40	0.85		
0.5	0	60	24.72	(1489)	+	1.17	(70)	+	0.28	(17)	+	26.17	(1576)	+	1563	1.28	32.33	31.77	66.66
0.0	0.025	60	7.25	(435)	+	4.15	(249)	+	1.32	(79)	+	12.72	(763)	+	728	2.34	31.52	31.15	86.71
Cross HB																			
0	0	60	0.78	(47)		0.17	(10)		0.00	(0)		0.95	(57)		57	2.00	1.95		
0.5	0	60	25.27	(1516)	+	0.85	(51)	+	0.12	(7)	+	26.23	(1574)	+	1622	1.24	32.64	30.89	67.16
0.0	0.025	60	6.82	(409)	+	2.58	(155)	+	0.73	(44)	+	10.13	(608)	+	595	2.13	22.31	20.37	89.96
mwh/TM3 (BH)																			
Cross HB																			
0	0	30	0.10	(3)		0.03	(1)					0.13	(4)		4	2.00	0.27		
0.5	0	30	9.03	(271)	i	0.23	(7)	i				9.27	(278)	-	278	1.18	10.78	10.54	
0.0	0.025	30	1.03	(31)	i	0.57	(17)	i				1.60	(48)	-	48	2.35	4.19	3.93	
*Cross HB*																			
0	0	30	0.33	(10)		0.03	(1)					0.37	(11)		11	1.36	0.48		
0.5	0	30	8.97	(269)	+	0.27	(8)	i				9.23	(277)	+	277	1.18	10.72	10.24	
0.0	0.025	30	1.37	(41)	+	0.37	(11)	i				1.73	(52)	+	52	1.71	2.91	2.45	

Marker-trans-heterozygous flies (*mwh/flr* ^*3*^ ) and balancer-heterozygous flies (*mwh/TM3*) were evaluated.

^a^ Statistical diagnoses according to Frei and Würgler (1988, 1995): +, positive; -, negative; i, inconclusive. m = multiplication factor for significantly negative results. Level of significance P ≤ 0.05.

^b^ Including rare f*lr* ^*3*^ single spots.

^c^ Considering *mwh* clones from mwh single and twin spots.

^d^ Frequency of clone formation: clones/flies/48,800 cells (without size correction) Frei *et al.* (1992).

^f^ Only *mwh* single spots can be observed in heterozygous individuals *mwh/TM3*, since the balancer chromosome *TM3* does not contain the mutant gene *flr* ^*3*^ .

### Carcinogenic effects

In a second moment, the ETT was conducted and, again, the TX test defined the range of concentrations to be evaluated. Toxicity was measured by the number of larvae exposed to CBP-01 that did not emerge after a chronic treatment of 48 h.

**Figure 2 -:**
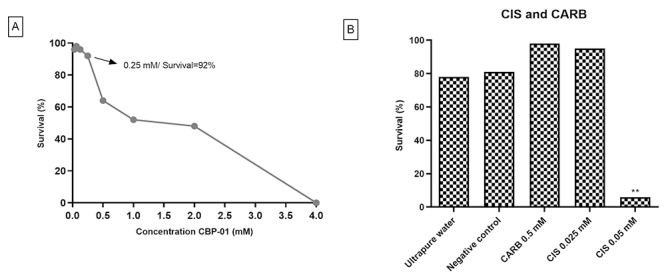
Survival rates of *D. melanogaster* upon exposure from third-stage larvae. (A) Larvae were treated with different concentrations of CBP-01. (B) Larvae were treated with different concentrations of carboplatin (CARB) and cisplatin (CIS). Larvae were obtained from crossing virgin *wts/TM3, Sb* ^*1*^ females with mwh/mwh males in the Epithelial Tumor Test - ETT. NC: Ultrapure water. **Statistical difference (p < 0.01) comparing to water control according to the X^2^ test for ratios for independent samples.

As with the SMART assay, a dose-dependent effect and a lethal dose of 4.00 mM were observed. The survival was over 90% at the concentration of 0.25 mM CBP-01 (Figure 2 A ), with no statistical difference when compared to negative control and ultrapure water. In the other concentrations, 0.03 mM, 0.06 mM, 0.12 mM and 0.25 mM, there was no significant difference when compared to negative control and ultrapure water. Again, the concentrations of CBP-01 chosen for further analysis were 0.03 mM, 0.06 mM, 0.12 mM and 0.25 mM.

CARB and CIS toxicity (Figure 2 B ) also followed the same pattern shown for the SMART test. Only 6% of adult individuals emerged from treatment of larvae with 0.05 mM CIS, which was statistically different from negative control and ultrapure water. 0.5 mM CARB and 0.025 mM CIS did not differ statistically from the negative control and ultrapure water, being non-toxic and therefore used in subsequent assays.

**Figure 3-:**
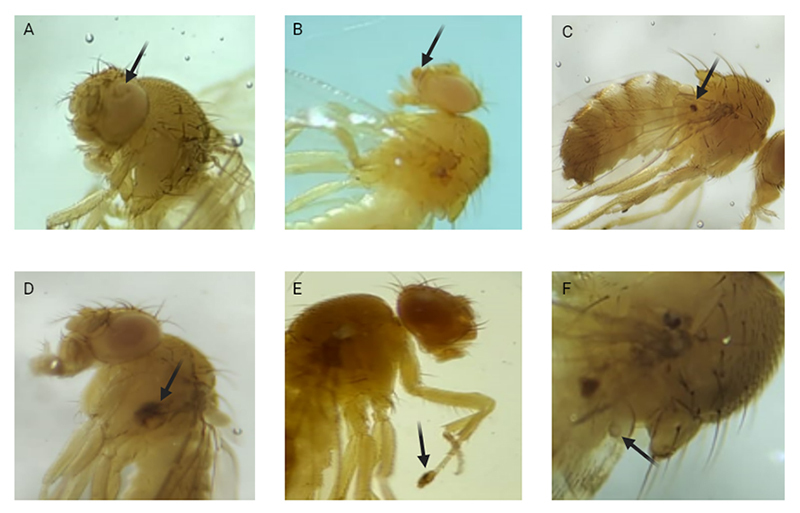
Tumors in different segments of *D. melanogaster* indicated by arrows. (A) tumor in the eyes. (B) tumor in the head. (C) tumor on the wing. (D) tumor in the body. (E) tumor on the legs. (F) tumor on the halters.

In ETT, flies of the (*wts +/+ mwh*) genotype were evaluated for the presence of epithelial tumor. Figure 3 shows tumors in different segments of the fly, which are quantified separately, according to the region analyzed.

Table 3 shows the frequency of tumors found in each segment of the adult fly after exposure of the larvae to different concentrations of CBP-01 (0.03 mM, 0.06 mM, 0.12 mM and 0.25 mM), CARB (0.5 mM), CIS (0.025 mM), DOX (0.4 mM, positive control) and negative control. No statistically significant difference was observed between the frequency of tumors in different concentrations of CBP-01 and the negative control, suggesting the absence of carcinogenic effect of CBP-01. DOX significantly induced the tumor frequency, and CARB and CIS showed a tumor frequency of 1.01 and 53.3, respectively, differing from the negative control.

**Table 3-:** Tumor clone frequency observed in *D. melanogaster*, heterozygote for the *Warts* tumor suppressor gene, treated with CBP-01 (0.03 mM, 0.06 mM, 0.12 mM and 0.25 mM)**,** carboplatin (CARB, 0.5 mM), cisplatin (CIS, 0.025 mM) and different concentrations of CBP-01 (0.03 mM, 0.06 mM, 0.12 mM and 0.25 mM) associated to CARB (0.5 mM). DOX (0.4 mM) was used as positive control and 5% ethanol as negative control. The frequency of tumors was analyzed in different segments.

Treatments		N^0^. of flies (N)	Spots per fly (n^0^. of spots) statiscal diagnosis^a^												Spots with mwh clone^c^ (n)	Mean clone size class^c,d^ (î)	Frequency of formation / 10^5^ cells per cells divisiond		Recombination (%)
			Small single (1-2 cels)^b^ m = 2			Large single (>2 cels)^b^ m = 5			Twin m = 5			Total spots m = 2					Observed	Control corrected	
CARB (mM)	CIS (mM)																		
mwh/flr3 (MH)																			
Cross ST																			
0	0	60	0.37	(22)		0.05	(3)		0.00	(0)		0.42	(25)		25	1.40	0.85		
0.5	0	60	24.72	(1489)	+	1.17	(70)	+	0.28	(17)	+	26.17	(1576)	+	1563	1.28	32.33	31.77	66.66
0.0	0.025	60	7.25	(435)	+	4.15	(249)	+	1.32	(79)	+	12.72	(763)	+	728	2.34	31.52	31.15	86.71
Cross HB																			
0	0	60	0.78	(47)		0.17	(10)		0.00	(0)		0.95	(57)		57	2.00	1.95		
0.5	0	60	25.27	(1516)	+	0.85	(51)	+	0.12	(7)	+	26.23	(1574)	+	1622	1.24	32.64	30.89	67.16
0.0	0.025	60	6.82	(409)	+	2.58	(155)	+	0.73	(44)	+	10.13	(608)	+	595	2.13	22.31	20.37	89.96
mwh/TM3 (BH)																			
Cross HB																			
0	0	30	0.10	(3)		0.03	(1)					0.13	(4)		4	2.00	0.27		
0.5	0	30	9.03	(271)	i	0.23	(7)	i				9.27	(278)	-	278	1.18	10.78	10.54	
0.0	0.025	30	1.03	(31)	i	0.57	(17)	i				1.60	(48)	-	48	2.35	4.19	3.93	
*Cross HB*																			
0	0	30	0.33	(10)		0.03	(1)					0.37	(11)		11	1.36	0.48		
0.5	0	30	8.97	(269)	+	0.27	(8)	i				9.23	(277)	+	277	1.18	10.72	10.24	
0.0	0.025	30	1.37	(41)	+	0.37	(11)	i				1.73	(52)	+	52	1.71	2.91	2.45	

Marker-trans-heterozygous flies (*mwh/flr* ^*3*^ ) and balancer-heterozygous flies (*mwh/TM3*) were evaluated.

^a^ Statistical diagnoses according to Frei and Würgler (1988, 1995): +, positive; -, negative; i, inconclusive. m = multiplication factor for significantly negative results. Level of significance P ≤ 0.05.

^b^ Including rare f*lr* ^*3*^ single spots.

^c^ Considering *mwh* clones from mwh single and twin spots.

^d^ Frequency of clone formation: clones/flies/48,800 cells (without size correction) Frei *et al.* (1992).

^f^ Only *mwh* single spots can be observed in heterozygous individuals *mwh/TM3*, since the balancer chromosome *TM3* does not contain the mutant gene *flr* ^*3*^ .

Larvae were also exposed to CBP-01 (0.03 mM, 0.06 mM, 0.12 mM and 0.25 mM) combined with CARB (0.5mM) (Table 3). The frequency of tumors found for all CBP-01 concentrations differed statistically (p < 0.05) from that found for treatment with 0.5 mM CARB alone. These results suggest a modulating effect of CBP-01 against damage induced by CARB. Therefore, the association of CBP-01 and CARB reduces the frequency of tumors, when compared to individuals treated with CARB alone. 

## Discussion

Tumor complexity and plasticity have limited the success of the therapies adopted, what requires the development of new, more assertive and effective strategies (Ji et al., 2023). CIS and CARB have been widely used to treat head and neck, cervical, ovarian, lung and testicular cancers (Ali et al., 2022; Pourmadadi et al., 2023). However, these compounds are toxic with lower cellular uptake and increased drug efflux (Rahiminezhad et al., 2022). Herein, we analyzed the biological effects of CBP-01 in *D. melanogaster* to validate its antitumor potential with lower mutagenicity/recombinogenicity. Copper has unique physicochemical characteristics and its remarkable biocompatibility makes it applicable to the medical field, especially oncology. In fact, copper concentration is capable of modulating tumor progression and may induce specific cytotoxicity (Aishajiang et al., 2023).

Firstly, the lethal dose of CBP-01 was determined and concentrations of 0.5 mM, 1.0 mM and 2.0 mM were toxic, reducing the percentage of survival when compared to the negative control. Copper, at high concentrations, can cause lipid peroxidation, oxidative stress, damage to proteins and DNA, mitochondrial dysfunction and cellular death, being potentially toxic to non-tumor cells (Chen et al., 2023). In *D. melanogaster,* the lowest concentrations of CBP-01 (0.03 mM, 0.06 mM, 0.125 mM and 0.25 mM) were nontoxic to descendants of the SMART and ETT tests, with survival rate up to 70% until 0.25 mM dose. These results demonstrate that CBP-01 was less toxic than other copper-based compounds such as copper(II) complex containing 4-fluorophenoxyacetic acid hydrazide and 1,10-phenathroline (Bontempo et al., 2022). 

In the SMART assay, CBP-01 was not potentially mutagenic / recombinogenic in ST cross, when compared to the negative control. However, a higher frequency of spots was observed in HB cross than in ST cross. Only at the lowest concentration of CBP-01 was the frequency of spots significantly higher compared to the negative control. The difference between HB and ST crosses is the P450 levels. ST-crossed flies present basal levels of this enzyme, which allows the evaluation of damages caused by direct action of genotoxins (Graf et al., 1984). HB-crossed individuals, in turn, have high levels of P450, identifying genotoxic damages of metabolites generated through the biotransformation of xenobiotics (Frölich and Würgler, 1989; Graf and van Schaik, 1992; Saner et al., 1996). We suggest that CBP-01, after metabolization, produced reactive substances, which interacted with DNA and led to a greater expression of mutant phenotypes. In fact, previous studies have indicated that the main mechanism of action of copper complexes involves the generation of reactive oxygen species (ROS) (Blackman et al., 2012; Graf and Lippard, 2012; Santini et al., 2014; Agbale et al., 2016; de Souza et al., 2019). Our group also demonstrated that a copper(II) complex with 4-fluorophenoxyacetic acid hydrazide and 1,10-phenanthroline promoted the production of ROS inducing DNA damage in sarcoma and melanoma cells (Machado et al., 2021).

The increase in mutant spots at the lowest concentration of CBP-01 (0.03 mM) was due to recombinogenic events (52.15%). In fact, increased ROS generation can lead to breaks in the DNA molecule, which can be repaired through the process of homologous recombination, favoring the expression of the mutant phenotype (Lahiguera et al., 2020).

In 2017, Serment-Guerrero et al. (2017) performed a DNA breakage test in bacterial cultures with Casiopeins (Cas III-Ea, Cas II-gly, Cas III-ia and Cas III-Ha) and found that these drugs caused different double-strand breaks (DSBs), probably due to oxidative damage. Cas III-Ea has completed preclinical trials and is ready to start clinical phase I in Mexico. Additionally, our group has already studied a similar ternary complex of copper(II) with doxycycline and 1,10-phenanthroline on somatic cells of *D. melanogaster* and we found that this compound significantly increased the frequencies of mutant cells in both ST and HB crosses, mostly through recombinogenic effect (Lopes et al., 2018). Interestingly, in this present study, when the concentrations of CBP-01 were increased, the number of spots decreased in both crosses. Thus, in the SMART test, as the concentration of CBP-01 increased (from 0.03 to 0.25 mM), damage may have also progressively increased leading to cellular apoptosis, reducing the expression of the mutant phenotype in the fly’s wing and resulting in lower frequency of spots without causing the lethality of the individual. We hypothesized that, with the increase in ROS production, defense mechanisms against oxidative stress were activated. In fact, in an earlier study, Jiménez et al. (2016) tested the synergism between the genotoxic and oxidative potential of Casiopeina II-gly, demonstrating that an increased drug concentration led to increased activity of the enzymes superoxide dismutase (SOD) and catalase (CAT). In this case, additional assays are needed to validate the suggested signaling pathways for CBP-01. 

In the ETT assay, none of the concentrations tested showed a carcinogenic effect. According to Vurusaner *et al.* (2012), ROS modulates the selective transactivation of genes, including tumor suppressors. Thus, the phenotypic effects observed reveal an orchestrated action between damage and cellular response, so that tumors were not observed in the segments of the flies. In addition, it is worth noting that the descendants of the ETT have basal levels of enzymes of the cytochrome P450 complex, different from the descendants of the HB cross evaluated in the SMART assay (Orsolin et al., 2012). 

Regarding the mutagenic agent used as positive control, we observed that DOX presented a significant frequency of spots, mainly induced by recombinogenic events. These results are in line with several studies with *D. melanogaster* and SMART, which reported the genotoxic effect of DOX and used this drug as positive control (De Rezende *et al.*, 2011; Machado et al., 2013; Orsolin et al., 2015; Silva-Oliveira et al., 2016; Oliveira *et al.*, 2017). Furthermore, the present data for the treatments with CARB and CIS alone corroborate previous results, in which the platinum-based compound was shown to be mutagenic / recombinogenic in *D. melanogaster* using the SMART assay (Danesi et al., 2010; de Campos et al., 2017).

Szikriszt et al. (2020) demonstrated that platinum analogues are mutagenic and CIS causes even more DNA damage than CARB, similar to what was found here. They further suggested that somatic mutations increase tumor heterogeneity and contribute to chemoresistance. Mutagenic chemotherapy drugs can also stimulate the formation of secondary tumors. This finding corroborates our data, since in the SMART test, both compounds (CARB and CIS) significantly induced the formation of mutant spots and, consequently, also showed a carcinogenic effect in the ETT test.

Zaidi et al. (2014) performed genotoxicity and oxidative stress tests *in vivo* comparing bis(1,2-diaminobenzene) copper (II)]chloride complex - CuSn_2_(Trp) to cisplatin demonstrating the potential of copper-based compounds and their promising properties when compared to drugs already incorporated in clinical practice. Although some researches make comparative studies reporting the greater cytotoxicity of the copper complexes, combined with the selectivity, to the platin analogues (Li et al., 2019; Szikriszt et al., 2020), few address the combined action of these compounds. Hence, our study is unprecedented and shows the modulating effect of CBP-01 on the carcinogenic action of CARB in *D. melanogaster*.

Two P-type ATPases ATP7A and ATP7B are well known for transporting copper into the cell. ATP7A is mainly expressed in the intestinal epithelium for copper absorption and its deletion causes systemic deficiency of the metal. The transporters, along with the high affinity copper transporter (hCtr1) and chaperone Cu (Atox1), are also involved in the transport of cisplatin and carboplatin. Furthermore, most, if not all, copper transporters are involved in the regulation of platinum chemosensitivity. In this context, targeting the copper transport system could be an effective approach to improving cancer therapy with platinum analogues. (Kuo et al., 2021). The copper transporters Ctr1A, Ctr1B, and Ctr1C are expressed in *D. melanogaster* and are codified by metallothionein genes, being induced by the transcription factor MTF-1 in response to the presence of metals. As in the tests we carried out the *D. melanogaster* larvae ingesting the compounds, we suggest a modulation in the copper receptors for the observed phenotypes. 

In summary, CBP-01 caused lower damages to somatic cells of *D. melanogaster* when compared to CARB and CIS and the interaction of CBP-01 with CARB reduced the number of tumors caused by the treatment with CARB alone. It should be noted that chemotherapy is a polypharmacological approach, where more than one drug is used in order to target cells at different stages (Kadu et al., 2021). Thus, CBP-01 modulates the genotoxicity and carcinogenicity of CARB, highlighting the benefit of this combination and opening a promising pathway in the determination of therapeutic regimens. Further assays should be conducted to validate the suggested mechanisms and other biological models should be used to confirm the potential of CBP-01 as antineoplastic drug.
